# Forced Wetting Properties of Bacteria-Laden Droplets Experiencing Initial Evaporation

**DOI:** 10.1021/acs.langmuir.3c00179

**Published:** 2023-04-20

**Authors:** Federica Recupido, Maria Petala, Sergio Caserta, Daniele Marra, Margaritis Kostoglou, Thodoris D. Karapantsios

**Affiliations:** † Division of Chemical Technology, School of Chemistry, Aristotle University of Thessaloniki, University Box 116, 54 124 Thessaloniki, Greece; ‡ Department of Civil Engineering, Aristotle University of Thessaloniki, University Box 10, 54 124 Thessaloniki, Greece; § Department of Chemical, Materials and Industrial Production Engineering (DICMaPI), Piazzale V. Tecchio 80, 80125 Naples, Italy; ∥ CEINGE Advanced Biotechnology, Gaetano Salvatore 486, 80145 Naples, Italy

## Abstract

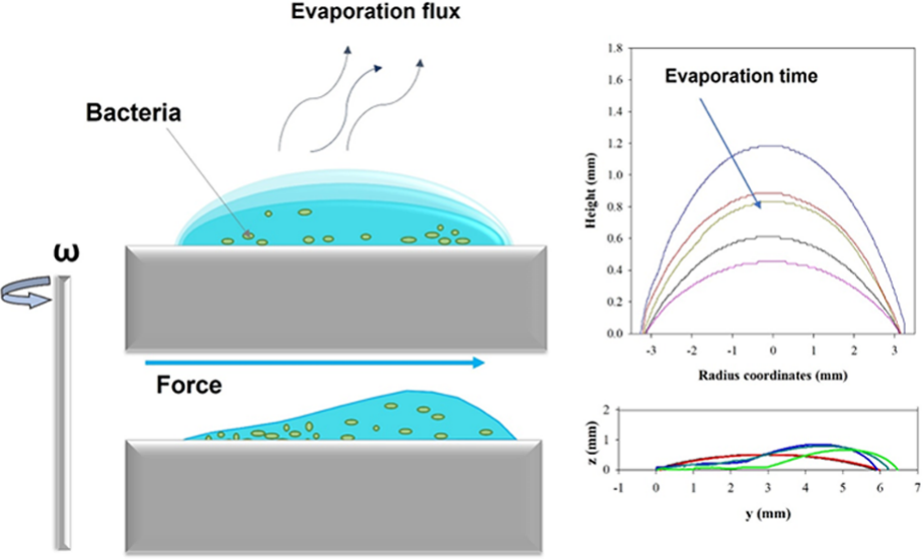

Microbial adhesion and spreading on surfaces are crucial aspects in environmental and industrial settings being also the early stage of complex surface-attached microbial communities known as biofilms. In this work, *Pseudomonas fluorescens*-laden droplets on hydrophilic substrates (glass coupons) are allowed to partially evaporate before running wetting measurements, to study the effect of evaporation on their interfacial behavior during spillover or splashing. Forced wetting is investigated by imposing controlled centrifugal forces, using a novel rotatory device (*Kerberos*). At a defined evaporation time, results for the critical tangential force required for the inception of sliding are presented. Microbe-laden droplets exhibit different wetting/spreading properties as a function of the imposed evaporation times. It is found that evaporation is slowed down in bacterial droplets with respect to nutrient medium ones. After sufficient drying times, bacteria accumulate at droplet edges, affecting the droplet shape and thus depinning during forced wetting tests. Droplet rear part does not pin during the rotation test, while only the front part advances and spreads along the force direction. Quantitative results obtained from the well-known Furmidge′s equation reveal that force for sliding inception increases as evaporation time increases. This study can be of support for control of biofilm contamination and removal and possible design of antimicrobial/antibiofouling surfaces.

## Introduction

Microbial adhesion and surface colonization represent crucial issues for either personal or public health. Microbial adhesion is also responsible for several industrial and environmental problems such as microbially induced contamination,^1^ contamination of the drinking water system,^2,3^ and cross contamination of food products.^4^ On the other hand, microbial spreading and attachment on solid surfaces represent the precursor of the formation of collective organizations known as biofilms^3,5−14^ whose implications are numerous in industrial and biomedical settings as well.^15−17^ Recently, bacterial growth, as well as biofilm contamination in space relevant conditions, have also been investigated.^18−20^

The biofilm formation process starts from the transport of free-living microbial cells and their initial adhesion on surfaces.^3^ This latter phenomenon was widely investigated according to thermodynamics and physical–chemical approaches. In the first case, contact angle measurements between liquids and solid substrates covered by bacterial lawns were used to assess microbial adhesion.^3,21^ In the second case, microbial adhesion was investigated by using the Derjaguin–Landau–Verwey–Overbeek (DLVO) theory, whereas the adhesion mechanism is governed by balancing electrostatic and van der Waals forces.^3^ In both cases, microbial cells are assumed to behave like inert colloids. During the step of cell adhesion in the surface contamination process by bacteria-laden liquids, the evaporation of the suspended liquid can take place. This aspect can be particularly relevant in the case of tiny droplets carrying microorganisms, where liquid evaporation can lead to relevant changes in cell concentration, affecting adhesion and subsequently the spread of droplets on surfaces. Typically, when the contact line of an evaporating droplet is pinned on the substrates during evaporation, a well-known phenomenon occurs (named as “*coffee ring effect*”), which is due to secondary convective flows induced by the Marangoni effect inside the droplet that leads to solute/particle deposition and accumulation at droplet edges.^22^ Marangoni flows are associated with surface tension gradients within the droplets, typically induced by either temperature or concentration gradients. Droplets carrying cells also show pattern depositions as a consequence of evaporation, with different patterns depending on the nature of microorganisms, e.g., presence/absence/type of motility,^23,24^ cell physiology,^25^ and metabolic activity. As an example, *Pseudomonas aeruginosa* bacterial cells produce biosurfactants, which promote the formation of a uniform ring on the substrates upon evaporation.^26^

*Escherichia coli* cells interact like sticky particles and form heterogeneous clusters which randomly adhere on surfaces affecting pattern deposition.^27^ In the case of evaporating droplets containing *E. coli* cells,^27^ concentration gradients of nutrients (i.e., sugars) drive bacterial chemotactic movement towards the nutrient source, inducing convective flows within the droplet and affecting deposition patterns. Understanding the microbial adhesion mechanism is fundamental to find strategies to prevent contamination. Although there is some literature about the fluid dynamics of evaporating bacteria-laden droplets, little is known about the interfacial properties of bacterial droplets on solid substrates and particularly how the evaporation affects wetting properties.

Wetting can indeed be successfully used to investigate interfacial properties of biofilm-coated surfaces,^12−14^ and it is in general considered as a powerful tool to investigate complex interfacial interactions, including bacteria-laden drops spreading onto clean surfaces.^11^ However, to the best of our knowledge, only a very few recent articles were focused on the wetting of bacteria-laden droplets, but never taking into account preliminary liquid evaporation.

In the work of Hennes et al.,^28^ spreading of *Bacillus subtilis* droplets on Agar substrates was investigated. Interestingly, it was found that bacteria exhibited collective behavior, inducing depinning and sliding of whole droplets, even at really small inclinations of the substrate. Also, other studies investigated the spreading of bacterial colonies on Agar substrates.^29,30^ Raj M et al.,^31^ investigated wetting properties of *E. coli* and inert micro-particle-laden droplets on superhydrophobic substrates (i.e., Teflon), evaluating both static and dynamic contact angles and adhesion forces by means of a custom-made cantilever device.

The present work investigates the wetting properties of bacteria-laden droplets interacting with hydrophilic substrates (glass), after initial evaporation of suspending medium having a primary effect on the densification of the bacterial load of the droplet.

*Pseudomonas fluorescens* AR11 strain is selected as the model system. This is a Gram-negative, rod-shaped aerobic bacterium, having several implications in the food industry; it is often associated with contamination of the diary industry facilities due to the short duplication times, resistance to heat treatments,^4^ and capability to form biofilms.^4,9,10,12−14^ In particular, the role of evaporation time on wetting properties of bacteria-laden droplets is systematically examined here for the first time. Evaporation/wettability tests are performed using a custom-made device named *Kerberos*(12,13,32−35) specifically designed to study the physics of static and forced wetting i.e., while external body forces such as gravitational or centrifugal ones are employed. *Kerberos* is also capable of achieving independent control of normal and tangential forces acting on sessile droplets, simultaneously performing tilting and rotation, and to monitor droplet shape deformation during motion in the three directions (*X*, *Y*, *Z*). Such a device was recently used to explore forced behavior of water droplets on biofilm-coated glass substrates developed under different growth conditions.^12,13^ In this work, only centrifugal forces are applied (no tilting). Droplets are subjected to evaporation before the wetting test. Retention forces for droplet onset for sliding (droplet depinning from the surface), representing an indirect measure of the bacterial adhesion force, are evaluated here as a function of different evaporation times by means of the well-known Furmidge′s equation, evaluating droplet retention force (*F*_S_),^33,36^ i.e., minimum force needed for droplet motion inception, as reported in eq 1
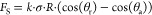
1where *F*_S_ is the retention force (N), *k* is the force retention factor, *R* is the characteristic length (m), and σ is the liquid surface tension (N/m) and cos(θ_r_) – cos(θ_a_) is defined as contact angle hysteresis, where θ_a_ is the maximum contact angle formed droplet that advances upon wetting the solid and θ_r_ is the receding contact angle formed when dewetting occurs.

The structure of the present work is as follows. In the first section, rheological characterization of bacteria-laden suspensions at different initial cell concentrations is presented. Subsequently, bacterial droplet evaporation is examined as a function of time and compared to that of the suspended medium. Live/dead cell assay is also performed on evaporating droplets to verify the actual state of microbial cells during the evaporation test.

In the last section, spreading behavior of partially evaporating droplets is investigated as a function of the applied rotation speed. Evaporation and subsequent spreading of droplets are examined by means of the basic features of static and forced wetting supported by a dedicated image analysis technique. The present work is crucial for understanding the initial stages of microbial adhesion on surfaces from precipitating droplets, e.g., from spillovers or splashing, where liquid evaporation occurs microbes′ concentration in the droplet increases, leading to preliminary stages of biofilm formation.

## Experimental Section

### Microorganisms and Culture Conditions

*Pseudomonas fluorescens* AR 11 cells (DSMZ-German Collection of Microorganisms and Cell Cultures) are cultivated in the sterile minimal medium supplemented with 0.1% glucose and in aerobic conditions as reported in previous work.^12^ Cells are grown at 30 °C overnight on an oscillating plate (80 rpm). Overnight-grown suspensions attain an optical density at 600 nm (OD_600nm_) of about 2 cm^–1^, which corresponds approximately to 3.9 × 10^8^ cells/mL and volume fraction (Φ) equal to 0.02%. After culturing, bacterial suspensions are centrifuged (Rotofix 32 A, Hettich Zentrifugen, Germany) at 10 000 rpm for 20 min and the supernatant is removed to get bacterial cells in the form of pellets. Pellets are resuspended in the minimal medium (with the same concentration used for bacterial culturing) to achieve a final OD_600nm_ of about 10 cm^–1^, corresponding to a highly dense bacterial suspension, with the aim to encourage bacterial accumulation. Resuspension with the nutrient medium is supposed to guarantee bacterial viability. Such a procedure is meant to mimic real situations where microbial droplets are spilled over onto surfaces, by subsequently adhering and forming biofilms.

To examine the effect of bacterial concentration on wetting properties, three different bacterial concentrations are examined corresponding to the following OD_600nm_ values: 2, 10, and 20 cm^–1^, respectively, which are obtained by resuspending cell cultures with different nutrient medium volumes.

### Rheological Characterization

Fluids rheology is characterized using a stress-controlled rheometer (Anton Paar Physica MCR 301) equipped with a coaxial cylinder cuvette geometry.^36^ Samples are covered by a thin film of low-viscosity silicone oil (Wacher Silicone Fluids, Germany) to avoid solvent evaporation. Measurements are carried out at 25 °C. Viscosity is measured as a function of shear rate (γ̇) in the range of 10^1^–10^2^ s^–1^, following the protocol previously validated for biological fluids and cell suspensions.^37,38^ After an initial pre-shear at γ̇ = 100 s^–1^ for 20 s to cancel loading effects, measurements are repeated changing the shear rate from low to high and back to low values. No significant hysteresis is observed. Nutrient medium and supernatant (i.e., solution obtained after centrifugation containing dissolved nutrients and exopolysaccharides produced by cell metabolism) are also characterized.

### Experimental Apparatus for Evaporation and Forced Wetting Tests

Tests on bacteria-laden droplets are performed using a custom-made device, named *Kerberos*, designed to study both static and forced wetting, where interfacial phenomena such as sessile droplet deposition on solid substrates, spreading, and sliding, are easily monitored in time by means of three wireless cameras (WCB-100A, Brickcom), equipped with 7× magnification lens (Olloclip, 3 in 1 Lens) observing the drop sample along *X*, *Y*, and *Z* orthogonal directions^12,13,32−34^*Kerberos* can perform a combination of tilting and rotation of the substrates where droplets are deposited on, by independently controlling tangential and normal forces acting on a sessile droplet.

In this work, *Kerberos* is used to perform two types of measurements: first to monitor droplet evaporation in time, in the absence of body force. After this step, forced wetting of partially evaporated bacterial droplets is observed, investigating the influence of different evaporation times. Bacterial suspensions are stained with very low volume (1:1000 dilution) of “*Brillant blue*” dye solution (0.5 g/L in bi-distilled water, Hina Dye Chem Industries) to enhance image contrast. It is verified that; such a low dye concentration does not alter the initial OD_600nm_ measure of the bacterial suspensions. Microscope slides (76.2 mm × 25.4 mm × 1 mm) are used as substrates for the wettability tests. Before the experiment, glass surfaces are cleaned according to the procedure described in our previous work.^12^

### Evaporation Tests

Evaporation tests are performed using a custom-made (82 mm × 30 mm × 22 mm) aluminum frame, installed inside the *Kerberos*′ rotating unit. A microscope slide is placed on the bottom part of the frame, while glass windows are installed around the lateral walls. The chamber allows ambient preconditioning achieving specific temperature and relative humidity conditions as reported in our previous works.^12,13^ A droplet of a fixed volume of 35 μL is gently deposited on clean glass through Eppendorf pipettes. Evaporation occurs under controlled air temperature and RH conditions (*T* = 25 ± 2 °C, RH = 50 ± 5%). Droplet evaporation is monitored by direct visualization using the three orthogonal cameras described above.

Acquisitions are done at defined time intervals i.e., every 5 min, at 10 frames per second. Short 20 s-long videos are stored in the.avi format for post-processing. The overall evaporation experiments last 60 min. Images of evaporating droplets at defined times are subsequently extracted and processed using a home-made Matlab code (v. 2013).^34^ The code provides automatically two-dimensional (2D) geometrical features such as contact angle, droplet length/height, and droplet shape/perimeter obtained from the analysis of the three views. The instantaneous droplet volume is estimated by fitting the experimental side profile of the droplet to the solution (numerical) of the axisymmetric Young–Laplace equation. The droplet shape must be described by this equation since the time scale of evaporation is much larger than the droplet hydrodynamic time scale. The required droplet volume is actually the fitting parameter at each time.

### Live/Dead Assay

To discriminate between alive and dead cells within evaporating droplets, a double staining procedure based on the live/dead viability assay (BacLight, Invitrogen, Carlsbad, California) is used. This kit consists of two fluorescent stains: the green dye SYTO 9 (3 μL/mL) and the red dye propidium iodide (1 μL/mL), enabling to stain alive and dead cells, respectively. Samples are incubated for 30 min in darkness with 2 μL droplets of each fluorescent dye.

Three-dimensional (3D) images of bacterial droplets are acquired using an inverted confocal microscope (LSM5 Pascal, Zeiss, Germany) equipped with helium/neon laser (LASOS Lasertechnik GmbH, LGK SAN7460A) using a 100 × oil-immersion objective (Achroplan, Zeiss) with 1.25 numerical aperture. The excitation/emission for selected dyes are 480/500 nm for the SYTO 9 stain and 490/635 nm for propidium iodide. *Z*-stacks images are acquired every 1 μm and recorded as tiff files.

The number of alive and dead cells during droplet evaporation is estimated through the image analysis procedure (Image Pro Plus 6.1, Media Cybernetics, Massachusetts). Images are split in the two channels (green and red) and segmented to estimate the number of bacteria, and a watershed filter was used to separate cell clusters. Cells are identified as isolated objects in the size range of 10–30 pixel^2^ (3.2–9.7 μm^2^). Average values ± standard deviation, calculated from at least triplicate independent experiments, are shown. Data are normalized with respect to the available surface (expressed as the number of cells/μm^2^). For each examined condition, three independent samples are analyzed and statistical significance is calculated using Student′s *t*-test (uncoupled, one tail). Differences are considered as significant for *p* < 0.005.

### Forced Wetting Tests

Wetting properties of bacteria-laden droplets are investigated using horizontal surfaces and applying exclusively centrifugal forces (no tilting). Rotation speed (RS) is increased from 0 with a ramp of 1 rpm/s up to the value of 100 rpm (rounds per minute). This range of RS corresponds to tangential acceleration varying from 0 to 27.4 m/s^2^*a*_T_ (*a*_T_ = ω^2^*r* where ω is the angular velocity ω = 2π*f*, *f* = RS/60 where *f* is the frequency in Hz, and *r* is the radial distance of the droplet from rotation axis ≅ 25 cm).

Experiments performed using droplets with no bacteria and only pure nutrient medium, are reported as negative controls. Wetting tests are carried out at specific times after droplet evaporation started. In particular, evaporation times of 0, 15, 30, 45, and 60 min are considered. Also, during rotation tests videos from the three views are recorded. Two-dimensional wetting parameters such as front and rear contact angles, droplet length, shape evolution and position of the droplet edges are obtained by extracting video frames from the side view. Droplet contours are obtained from the top view. All data are reported as a function of the instantaneous rotation speed.

For each evaporation time, the tangential dimensionless bond number (Bo_T_) for bacterial droplet onset for sliding from the surface is obtained as follows
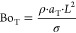
2where ρ · is the liquid density (kg m^–3^), *L* (m) is the characteristic length, *a*_T_ is the tangential acceleration (m/s^2^), and σ is the surface tension (N/m). As characteristic length *L* = 1 mm is assumed as characterizing the droplet dimensions according to that reported elsewhere.^35^ Such a dimensionless number is meant to correlate external body forces with surface tension forces acting on single droplets.

## Results and Discussion

Experimental data of evaporation and forced wetting tests are reported in this section. First, preliminary characterization of the examined wetting agents is provided in terms of rheological properties. Subsequently, a general description of the basic physics of droplet evaporation is elucidated. Finally, forced wetting of evaporating droplets is analyzed, reporting front and rear contact angles in addition to droplet length and contours.

### Liquid Characterization

**Figure 1:**
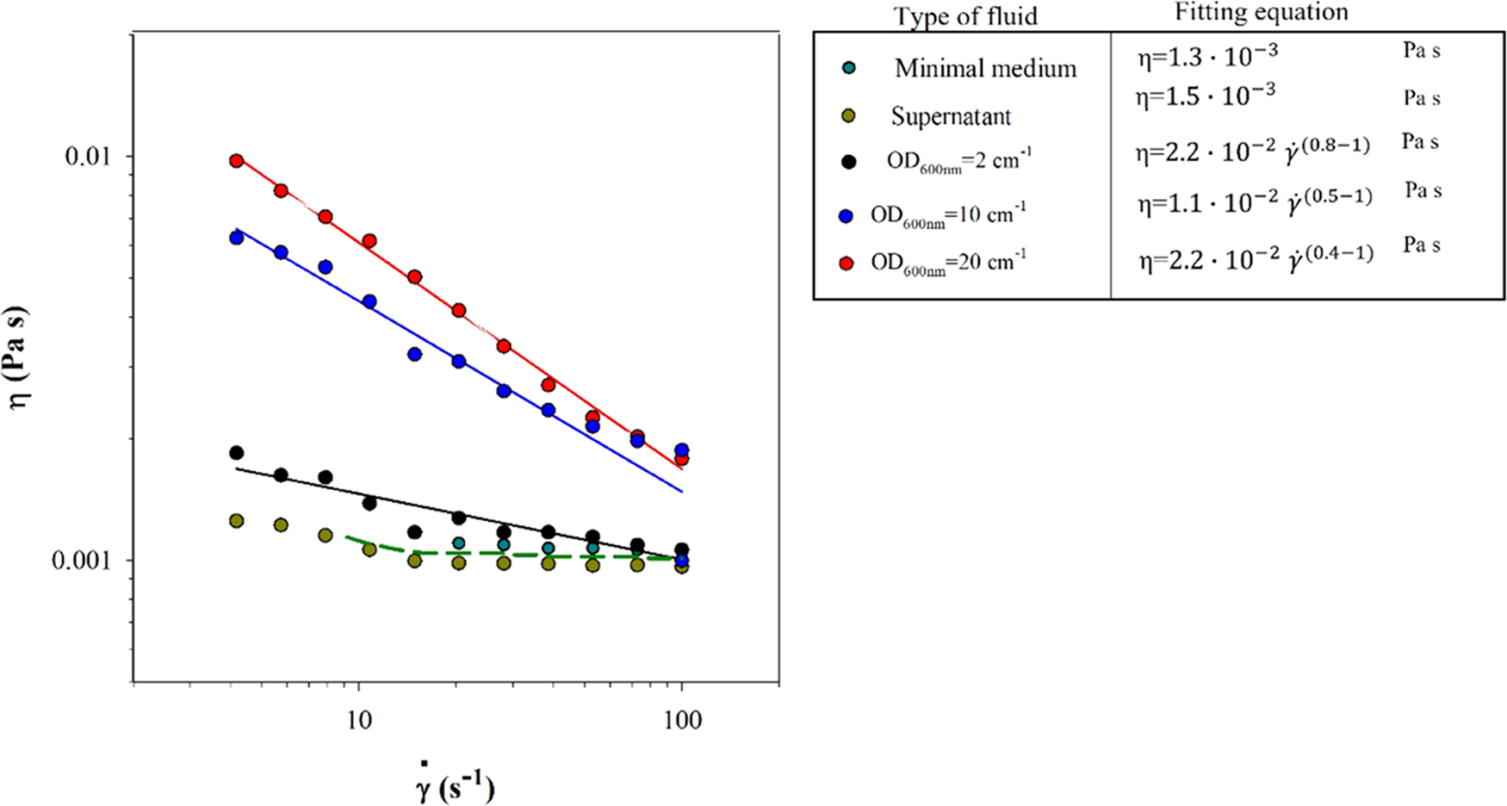
Apparent viscosity against the shear rate for bacteria-laden droplets at different cell concentrations reported as OD_600nm_. A comparison with the nutrient medium (i.e., suspending medium) and the supernatant (i.e., the solution obtained after centrifugation containing dissolved nutrients and exopolysaccharides produced by cell metabolism) is performed.

Apparent viscosity is assessed as a function of the initial bacterial concentration. Figure 1 reports viscosity as a function of the shear rate (γ̇) for three bacterial concentrations, corresponding to OD_600nm_ = 2, 10, and 20 cm^–1^, respectively. A comparison with minimal medium and supernatant solutions is also reported.

The viscosity shows a typical shear thinning behavior for the three examined bacterial concentrations. Fitting of the power law (η = *k*γ̇^*n*–1^) is reported in the figure legend.

The minimal medium and supernatant show a Newtonian behavior, maintaining the viscosity variation with the shear rate within the instrument sensitivity (average viscosity is shown in the figure legend). Low-density bacterial suspension (OD_600nm_ = 2 cm^–1^) also has limited thinning (*n* ≅ 0.8). In the case of higher cell density, higher viscosity values are measured, with a significant shear thinning behavior (*n* ≅ 0.5 and 0.4 for OD_600nm_ = 10 cm^–1^ and OD_600nm_ = 20 cm^–1^, respectively).

This trend is in agreement with that observed for *P. aeruginosa*(39) and *Pseudomonas putida*.^40^ A possible reason for such behavior can be ascribed to shear-induced alignment or breaking of cell aggregates, or eventually to cell deformation as reported in refs (37) and (41).

### Evaporation Tests

The evaporation of sessile droplets laden with microbial suspensions is investigated. Droplets on glass substrates undergo evaporation for different time lags (up to 60 min). Droplet evaporation is analyzed every 5 min, measuring macroscopic parameters such as left and right contact angles, droplet length, and height, as well as droplet shape, as a function of time. A comparison with the droplets containing only the nutrient medium is also reported.

**Figure 2:**
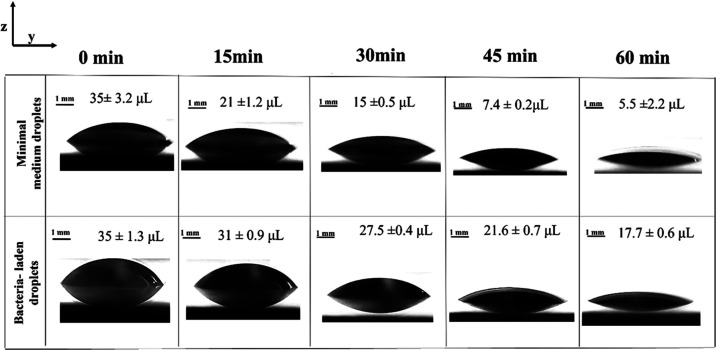
Sessile droplet evaporation in time for minimal medium and bacteria-laden droplets (roughly indicated as initial OD_600nm_ = 10 cm^–1^). Images are taken at subsequent evaporation times (i.e., 15 min interval). 7× magnification, scale bar = 1 mm.

In Figure 2, qualitative investigation of droplet shape evolution with evaporation time from side view for bacteria-laden and for minimal medium droplets (negative control), is reported in the top and bottom line of images, respectively. Images are reported at a time interval of 15 min. For this analysis, it is decided to consider the case of bacterial droplets having an initial OD_600nm_ value of 10 cm^–1^.

Qualitative estimation of the droplet contours during the evaporation tests shows that bacteria-laden droplets present lower evaporation rate compared with the negative control ones. For each case, the average and standard deviation (SD) of droplet volume is reported as inset.

**Figure 3:**
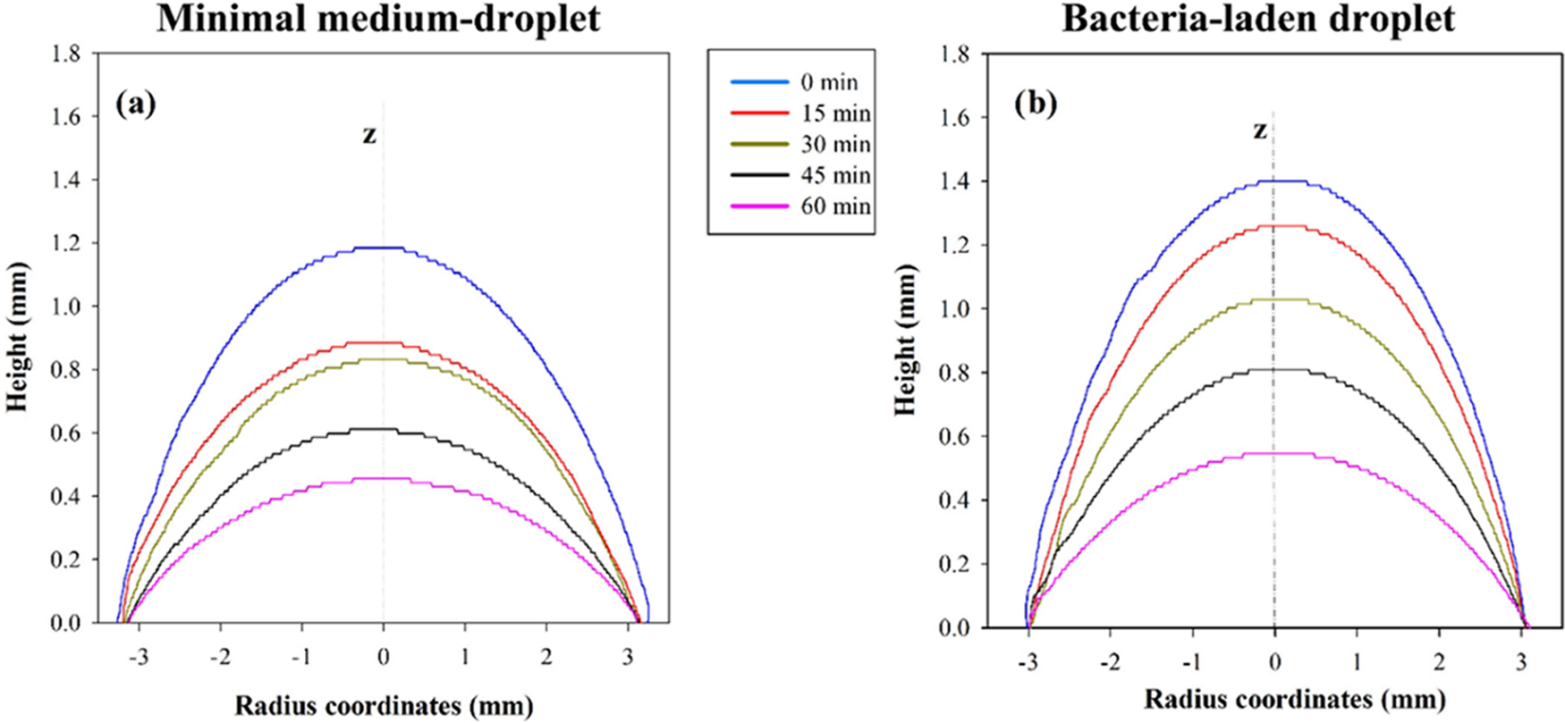
Experimental droplet contours parametric in the evaporation time (measured every 15 min) for (a) the minimal medium (negative control, no bacteria) and (b) the bacteria-laden droplet (initial OD_600nm_ = 10 cm^–1^).

To support these preliminary observations, droplet shape evolution is measured by analyzing side-view video frames obtained during the evaporation tests. Edge profiles of images, reported in Figure 2, are provided in Figure 3, where on the vertical axis the height of the droplet profile is reported as a function of radial coordinate for different evaporation times. The droplet length can be estimated as the difference between positions of the two edges. The droplet shape does not always maintain a strict axial-symmetric shape during evaporation. For minimal medium droplets (Figure 3a), the droplet depins soon after 15 min, showing a faster reduction of the droplet height with respect to those containing microorganisms (Figure 3b). Moreover, minimal medium droplets do not keep axial symmetry during the evaporation, while bacterial droplets do, remaining pinned on both sides for almost the entire duration of the test (60 min).

**Figure 4:**
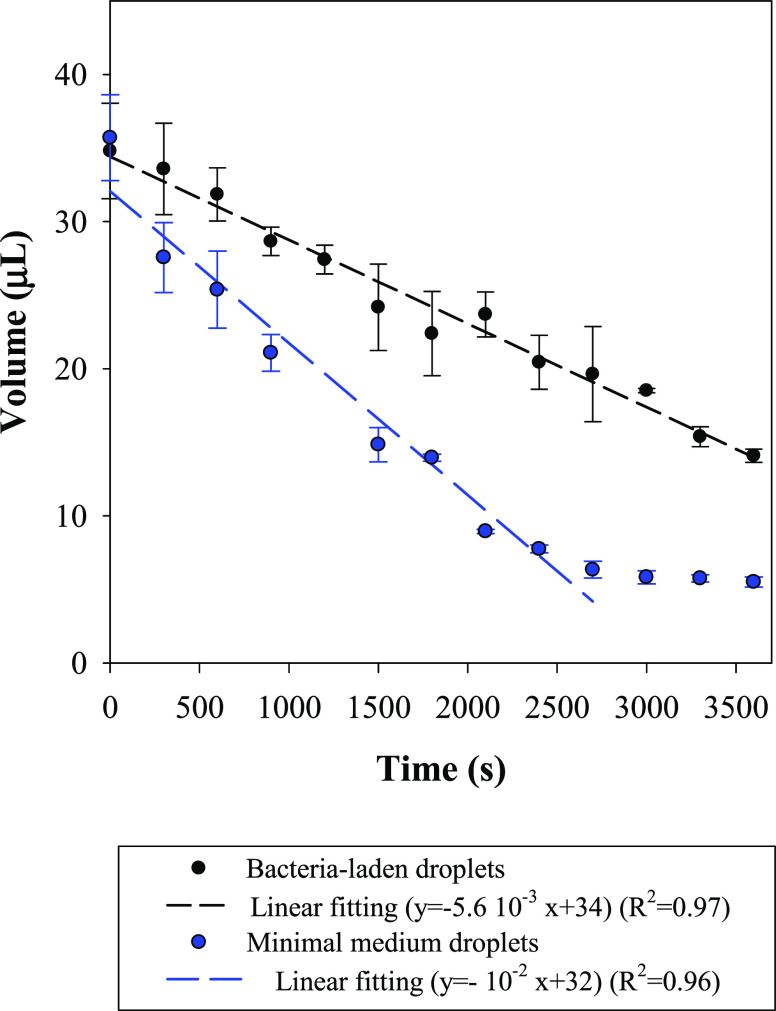
Droplet volume estimation as a function of evaporation time for minimal medium (blue curve) and bacteria-laden droplets (initial OD_600nm_ = 10 cm^–1^, black curve). Evaporation is monitored every 5 min. Data are reported as the average and standard deviation of three independent measurements. For both the two systems, the linear fitting of the experimental data is displayed along with *R*^2^.

In Figure 4, the evolution of the droplet volume, estimated by employing Young–Laplace equation as previously discussed is shown as a function of time (in seconds). Such analysis is meant to provide a more quantitative evaluation of the droplet evaporation rate in time, i.e., evaporating mass flux. Data report the average and error bar standard deviation calculated from three independent measurements. Fitting lines (Figure 4, dashed lines) and their relative equations along with mean-squared error (*R*^2^) of each set of experiments are also displayed.

In the case of the minimal medium, the droplet volume rapidly decreases (Figure 4 blue curve), down to a volume of 5.5 μL at the end of the experiment. A faster decrease is observed in the first 30–35 min, while a limited decrease is observed in the last step. Droplet volume vs time can be reasonably approximated by a linear function (Figure 4, blue dotted line, *y* = −10^–2^*x* + 29.9, *R*^2^ = 0.96) up to 50 min (3000 s), whereas, at larger times, constant volume can be noted, driving the system away from being linear. This might be ascribed to the fact that, after 50 min, a consistent amount of liquid evaporates (7 times less than the original droplet volumes), which clearly produces some experimental errors.

For bacteria-laden drops, volume linearly reduces in time (Figure 4, black curve) with a lower rate compared to the case of minimal medium i.e., 50% less than that of the minimal medium in terms of slope (5.6 10^–3^ vs 10^–2^) with *R*^2^ (0.97). This verifies that the droplet volume of bacteria-laden droplets can be approximated through a linear fitting equation. Therefore, at this stage, it can be concluded that despite some experimental errors, droplet volume vs time exhibits a linear trend under the investigated conditions. However, it must be stated that, in the literature, the evolution of the droplet volume with time is conventionally estimated according to a power law function,^42^ and the following findings are meant to merely provide evidence of differences in terms of evaporation velocity among the two examined systems.

The dissimilarities observed can be attributed to differences in the driving force leading to evaporation. In fact, the presence of bacteria affects the chemical potential and the fugacity of water, with respect to the case of the minimal medium. Differences in fugacity also induce different contact angles and droplet shapes. For this reason, the impact on the evaporation rate is more difficult to quantify, also dependent on effective areas at liquid–gas interfaces. It must also be pointed out that the presence of active matter (i.e., suspended cells) makes the scenario even more complex, as it can significantly alter the chemical potential of the system, due to combination/competition among passive (Marangoni effect) and active transports (cell chemotaxis/bacterial motility), which makes the evaporation a non-trivial process. Conclusively, the evolution of the evaporating droplet is hence investigated from a phenomenological point of view, whereas, considerations of a mechanistic approach are beyond the scope of this work.

**Figure 5:**
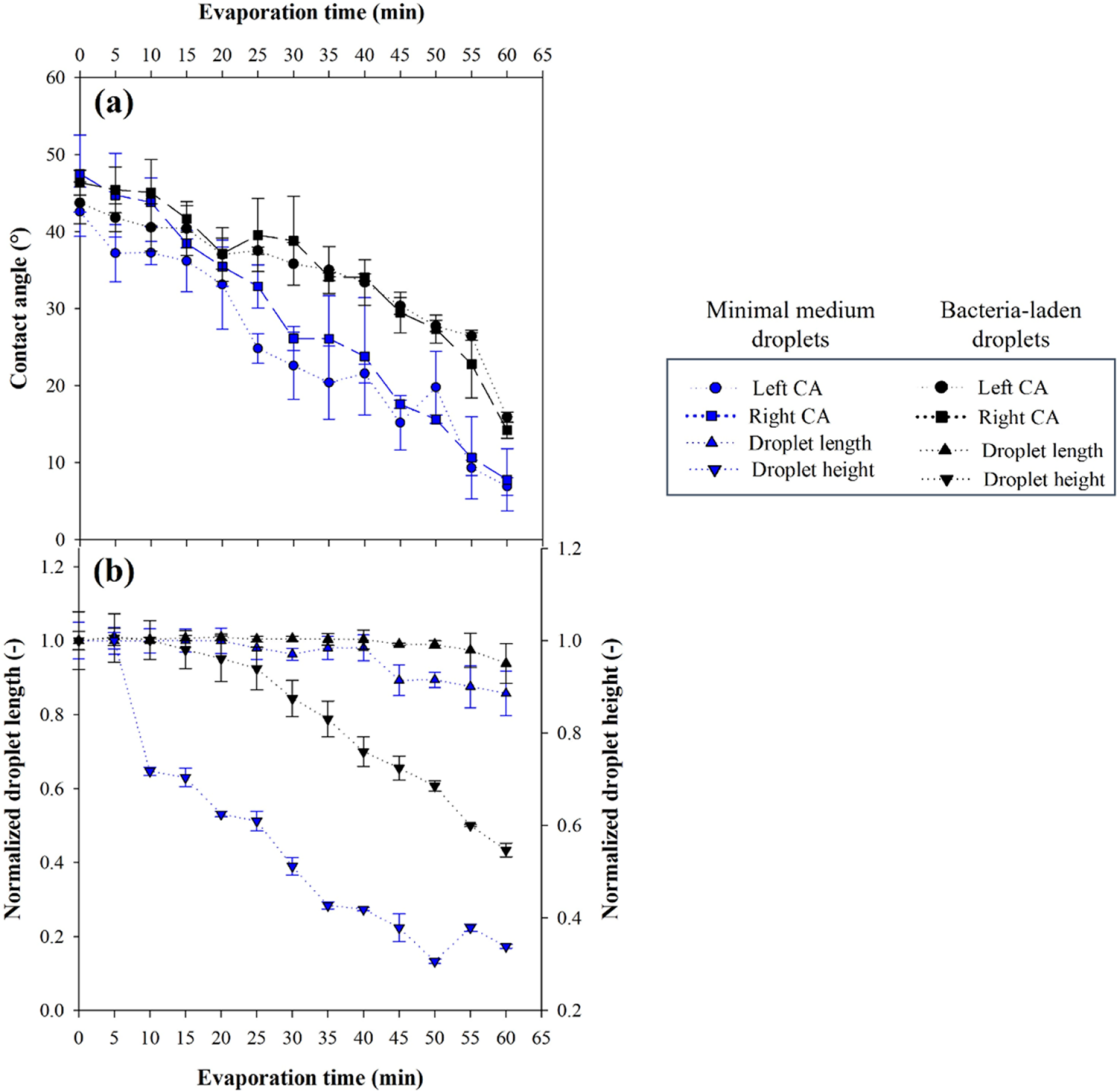
(a) Left and right contact angles and (b) normalized droplet length and height vs evaporation time for bacteria-laden droplets corresponding to an initial OD_600nm_ = 10 cm^–1^. Negative control of the experiments are droplets containing only the minimal medium (blue dots, without bacteria). Experiments are reported as the average and standard deviation of three independent measurements. In (b) droplet height vs time is shown on the right vertical axis.

The evolution in time during evaporation of the left and the right contact angles (a) and normalized droplet length/height (b) of concentrated bacteria-laden droplets (initial OD_600nm_ = 10 cm^–1^) and minimal medium droplets are shown in Figure 5. Experimental results are reported as the average and standard deviation of three independent measurements. This analysis is crucial in order to achieve a complete understanding of the evaporation process, as it might effectively induce variation of either droplet contact angles or droplet length or, in more complex scenarios, both of them, as reported by ref (42).

Bacteria-laden droplets show comparable initial contact angle values with respect to the minimal medium with an average contact angle of 40° (Figure 5a). The hydrophilic behavior observed is comparable to that observed in the case of pure water on biofilm-coated glass^9,12,13^ and on bacterial dehydrated lawns.^22^

Contact angles are significantly reduced after 30 min, attaining a final average value of about 20° at the end of the test for bacteria-laden droplets (Figure 5a, black dots). As a consequence, droplet height reduces in time and once a significant amount of liquid is lost, droplet retraction along length the direction is observed (Figure 5b, black dots), inducing contact line depinning. Nevertheless, the normalized droplet length remains constant and slightly drops soon after 40 min (Figure 5b, black dots). This suggests that the contact line remains pinned throughout the test. In the case of the control sample, contact angles have a similar initial value, but progressively reduce faster in time achieving a very small average value at the end of the evaporation test (about 7°, Figure 5a, blue curve). Normalized droplet length (Figure 5b, blue curve) starts decreasing after 30 min achieving a reduction of 16% with respect to the initial length at the end of the evaporation tests. At the same time (30 min) a change is observed in the reduction trend in the measurement of the droplet height (Figure 5b, blue down triangles).

Furthermore, with respect to the case of pure water, the evaporation rate should be reduced as water salinity increases, which leads to a decrease in the saturation vapor pressure of the solution at the given temperature, as has been reported.^43^ The reason for the different evaporation rates among bacteria-laden and minimal medium droplets might be ascribed to the production of biosurfactants from bacterial cells, which actually discourages evaporation by affecting water fugacity, as well as pattern depositions.^21−25,44−46^ As in the case of colloidal suspensions, the formation of specific coffee ring patterns depends on contact angle as numerically and experimentally demonstrated.^47−50^ Moreover, bacteria deposition at the edges may be also hindered by a nutrient-rich medium resulting in orientations of individual cells within the droplet bulk.^50^ Similar considerations are also reported for the case of droplets containing insoluble surfactants without bacteria.^51^

On the other hand, minimal medium droplets contain solutes such as glucose and salts (predominantly phosphates and chlorides). It is believed that in this case, solubility-induced Marangoni stresses is obtained leading to salt crystallization and accumulation near the contact line as investigated by the recent literature.^43^ Among the available literature, it is worthy to compare the present experimental data with numerical investigation shown in ref (45) where evaporation of bacterial cells and subsequent infiltration/retention within the leaf surface has been thoroughly studied. The authors stated that the evaporation phenomenology is complex and different processes (i.e., gas transport, fluid flow, cell chemotaxis, production of biosurfactants, and so forth) can simultaneously occur. As a main outcome, higher evaporation rate, translating into higher temperature gradient among the inner part and the contact line of the droplet (fugacity of the system depends on partial pressure and on the temperature) is associated with higher hydrophilicity (i.e., lower contact angles), which leads to bigger droplet depinning.^45^ So, this may be the main reason why, medium droplets, which possess higher hydrophilicity, evaporate faster than those containing bacteria (in addition to the already-mentioned aspects associated with bacterial motility).

**Figure 6:**
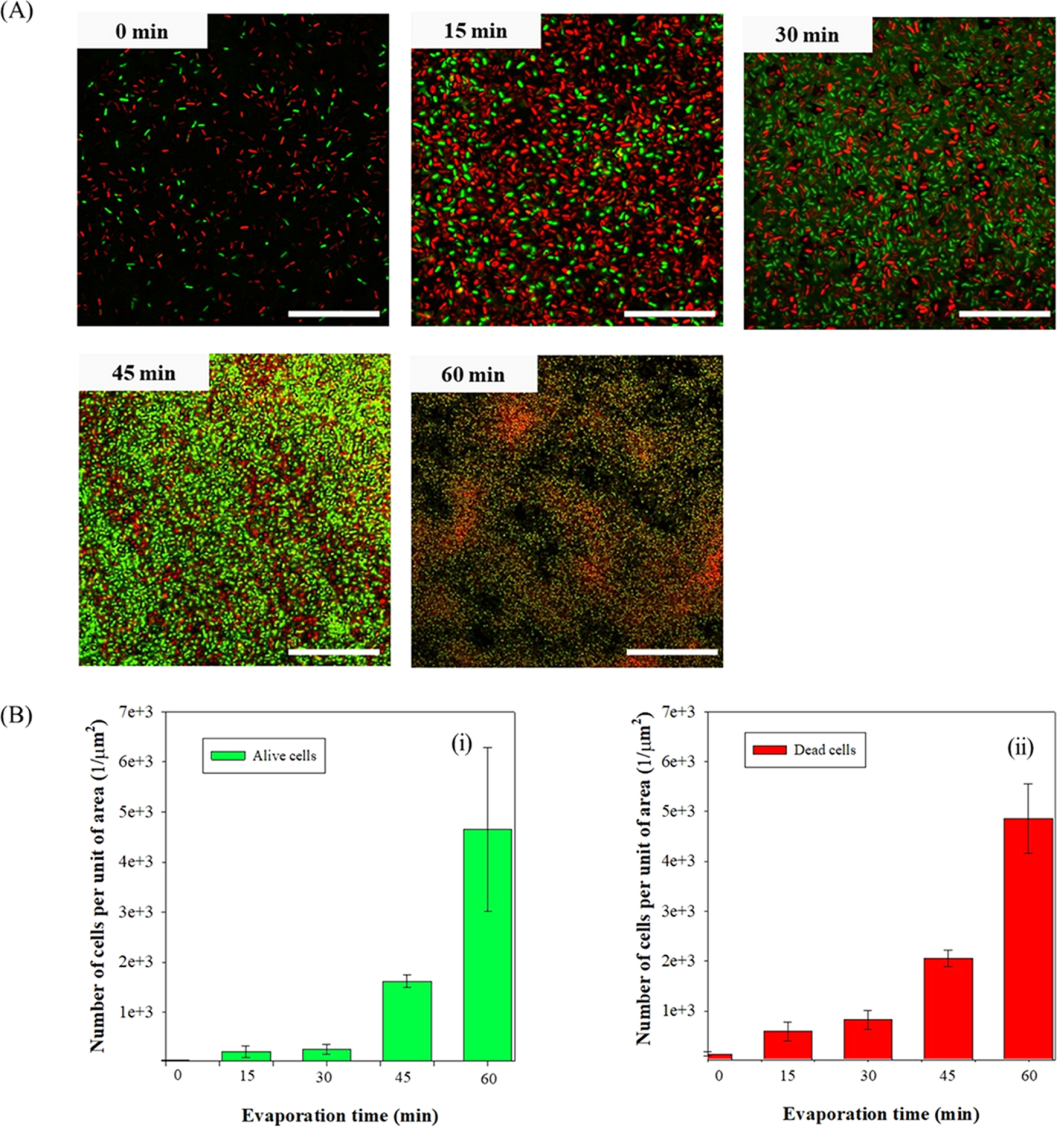
(A) Confocal stack images of bacteria-laden droplets (initial OD_600nm_ = 10 cm^–1^) containing alive and dead cells, at 0, 15, 30, 45, and 60 min-evaporation time. 100 × oil-immersion magnification. Scale bar = 20 μm. (B) Number of alive (i) and dead cells (ii) is shown as a function of the evaporation time.

The actual state of bacterial cells along the evaporation process is also analyzed here as the different natures of microbial cells strongly affect wetting characteristics as reported by ref (30). In Figure 6A, the maximum projection of *z*-stack merged images of bacterial droplets at the selected evaporation times is presented. In Figure 6B, the number of alive (i) and dead cells (ii), is estimated at the selected evaporation times.

The number of bacterial cells increased as a function of the evaporation time for both populations (Figure 6A). As the evaporation proceeds, denser agglomeration of cells within droplets is identified. At all investigated times, bacterial cells within evaporating droplets exhibit rod-shaped morphology, although, after 45 min, cells get smaller, perhaps due to stress conditions such as starvation, lack of nutrients, or low moisture or water depletion stress.^50^ No significant variations are attained at higher times (60 min).

As demonstrated in Figure 6B live cells keep almost a constant number up to 30 min, while they show a net increment after 45 min. On the other hand, the number of dead cells increases rapidly in time (Figure 6B,ii), up to 45 min, where an increase of 1 order of magnitude is noticed in the range of 30–45 min. This might be due to the fact that under the examined time window, droplets started to be consistently dried out leading to an increase of salt concentration (especially NaCl), which is translated to lower cell viability.^44^

After that period, in the last time window (45–60 min), both cell numbers remain almost constant, within the error bar (*p* < 0.005). Overall, for longer evaporation times, the number of alive cells is comparable to that of dead cells. The obtained results can be compared with the work of Liang et al.,^52^ where the effect of air RH as well as of bacterial concentration on cell inactivation is investigated. The authors stated that the population effect is crucial as it potentially reduces the osmotic stress, impeding cell inactivation during the evaporation.

### Forced Wetting Tests

After specified times of partial evaporation, sessile droplets are subjected to centrifugal forces using *Kerberos* to investigate forced wetting. Experiments are carried out using a target rotation speed of 100 rpm with a speed rate of 1 rpm/s. The selected procedure of forced wetting permits to maintain the normal forces acting on droplets constant, by monitoring the tangential forces, which gradually increase during the experiment. This is not possible performing tilting, where also the normal forces can vary and larger accelerations than 1 g cannot be attained.

**Figure 7:**
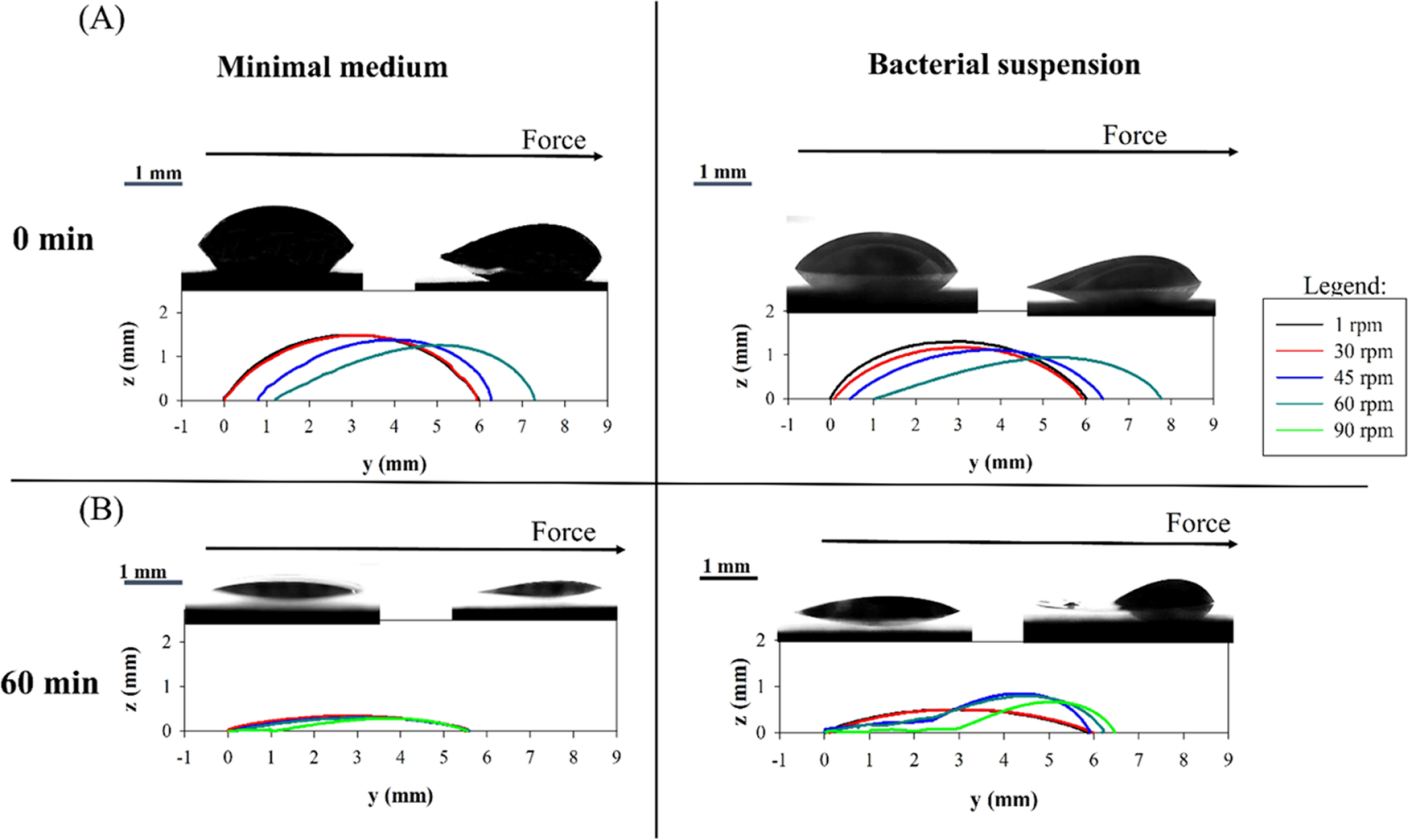
Side view contour evolution of the nutrient medium and bacteria-laden droplets (initial OD_600nm_ equal to 10 cm^–1^) at different evaporation times: (A) 0 min (no evaporation) and (B) 60 min. Droplet contours are obtained at consecutive rotation speeds. For each condition, images of droplets at the first and the last rotation speed values are embedded. Scale bar = 1 mm.

**Figure 8:**
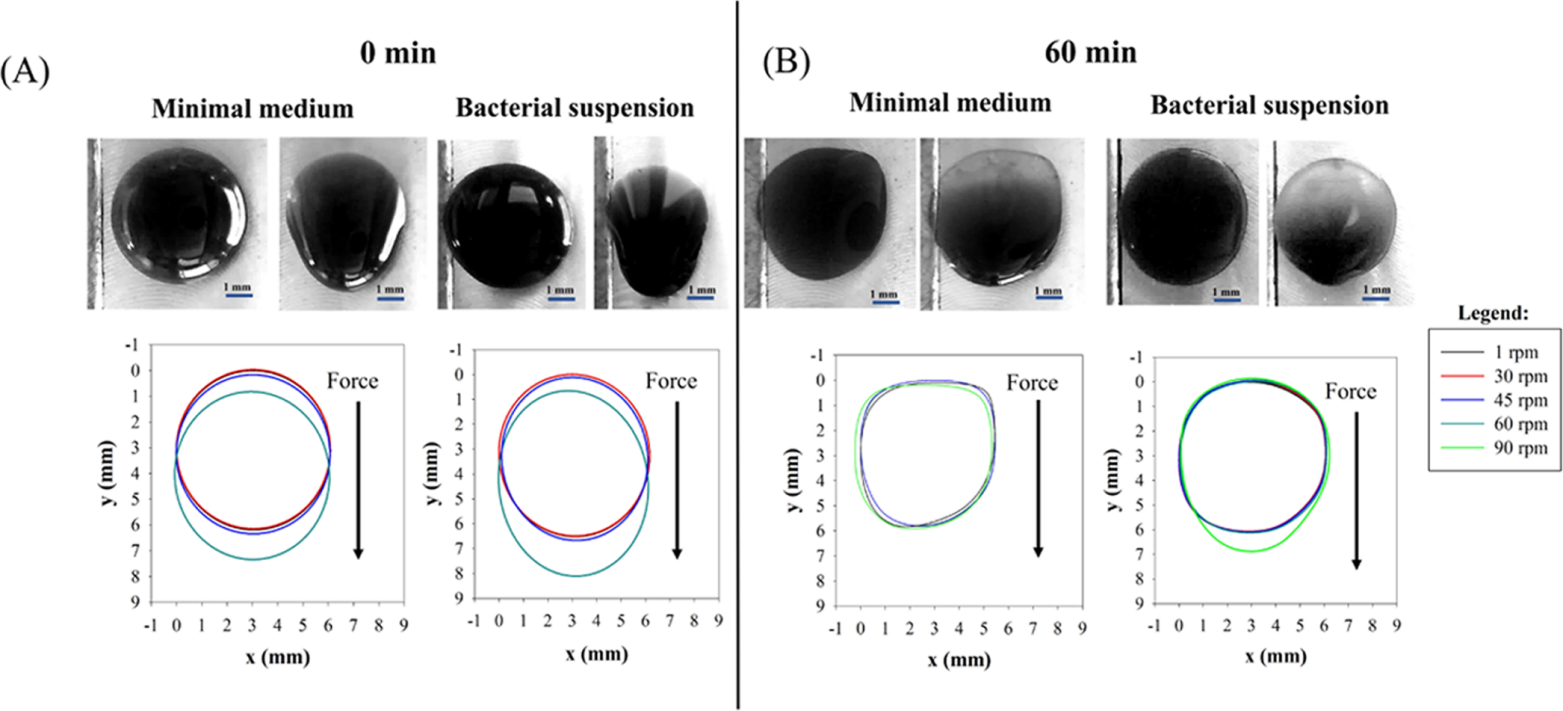
Side view contour evolution of nutrient medium and bacteria-laden droplets (initial OD_600nm_ equal to 10 cm^–1^) at different evaporation times: (A) 0 min (no evaporation) and (B) 60 min. Droplet contours are obtained at consecutive rotation speeds. For each condition, images of droplets at the first and the last rotation speed values are embedded. Scale bar = 1 mm.

In Figure 7 droplet shape evolution as a function of the rotation speed is reported for the two samples investigated i.e., minimal medium and bacterial suspensions having initial OD_600nm_ = 10 cm^–1^. The behavior of non-evaporated fresh droplets (Figure 7A, 0 min) is compared to the case of partially evaporated droplets (Figure 7B, 60 min). Upper panel of Figure 7A,B presents droplets in the first (0 rpm) and the last frames (60 and 90 rpm for non-evaporated and partially evaporated droplets, respectively), while, in the lower panel of Figure 7A, droplet contours at specific rotation speeds are shown. In Figure 8, the analysis is reported for the same droplets, as observed from top view. For both the examined cases of non-evaporated droplets (Figures 7A and 8A), the shape evolution is comparable with that of water on pure glass,^32^ i.e., droplets first spread and then sliding occurs at higher rotation speeds. A higher elongation is observed in the last steps of the experiment (for higher rotation speeds) for bacteria-laden droplets with respect to the negative control.

In the case of 60 min-evaporated droplets (Figures 7B and 8B) at higher rotation speeds (above 60 rpm), bacterial droplets show two different regions: a dark bulky front region (right edges of the image, where most of the liquid accumulates dragged by the centrifugal force) and a tail that remains visible on the rear part (left side of the image) where dried bacterial cells are mainly accumulated after evaporation (images are embedded in Figures 7B and 8B). Evaporated bacteria-laden droplets elongate advancing in the direction of the force, but never depin their edge on the opposite side (rear).

This peculiar presence of 2 different regions, is comparable to what was observed in the case of pure water droplets on mature biofilm-coated surfaces as reported in our previous works.^12,13^ In the case of partially evaporated nutrient medium droplets, this phenomenon is not observed. From the top view (Figure 8A,B), droplets show initial smooth and spherical contours, whereas elongated shapes are induced by deformation at higher rotation speeds.

**Figure 9:**
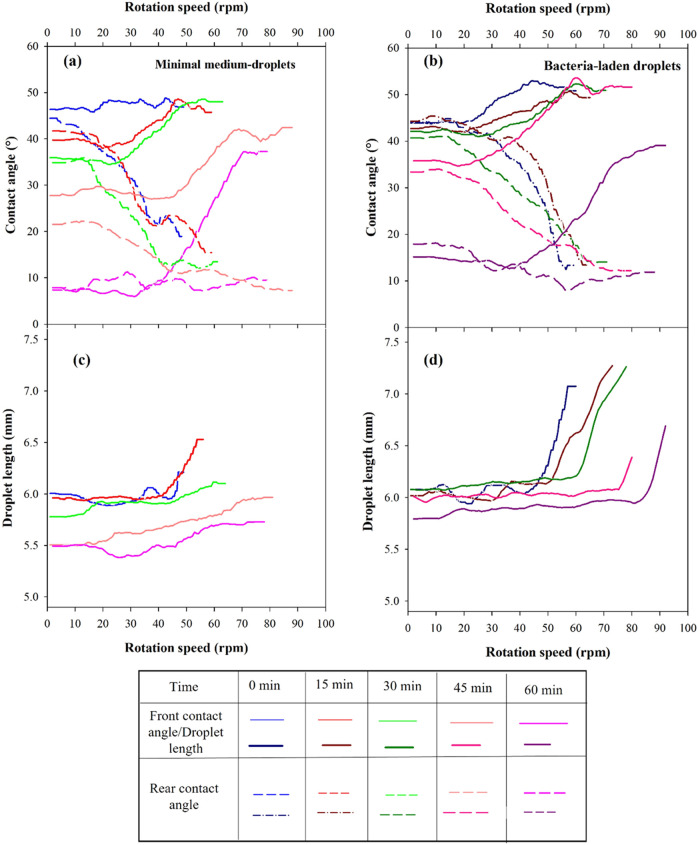
Front (straight lines) and rear contact angles (dotted lines) of the nutrient medium (a) and bacteria-laden (initial OD_600nm_ = 10 cm^–1^) (b) as a function of rotation speed at defined evaporation times. Droplet length vs rotation speed at defined evaporation times for nutrient (c) and bacteria-laden droplets(d), respectively. Wetting properties are investigated every 15 min up to 60 min of evaporation. Rotation speed is increased up to 100 rpm using an increase rate of 1 rpm/s.

Detailed analysis of forced wetting is reported in Figure 9 in terms of front and rear contact angles as well as droplet length as a function of the rotation speed for different evaporation times. Front and rear contact angles are shown in Figure 9a,b for nutrient and bacteria-laden droplets, respectively. Droplet length vs rotation speeds is displayed on different panels for the case of nutrient medium (Figure 9c) and bacteria-laden drops (Figure 9d). Data are reported for one droplet for each sample type, to appreciate differences in terms of forced wetting properties of the examined wetting agents by varying the evaporation time. Reproducibility of the measurement is reported in Figure S1 of the Supporting Information, where front and rear contact angles and droplet length for the case of bacteria-laden droplets for two selected different evaporation times of 30 min (A) and 45 min (B), are reported for three independent measurements. The evaporation time plays an important role in modulating forced wetting properties. First of all, evaporation determines a decrease in the initial droplet contact angles and length for both the examined wetting agents.

For a specified evaporation time, as the rotation speed increases, the front contact angle progressively increases along the centrifugal forces, whereas the rear contact angle decreases for both the examined liquids. As the rotation speed reaches a critical value, the front contact angle attains a constant value defined as advancing contact angle, θ_a_, while a rear contact angle continues decreasing. This corresponds to the movement of the front edge and the start of droplet spreading. As the rotation speed increases, the front edge still moves with the advancing contact angle, while the rear one reaches a final contact angle value defined as the receding contact angle (θ_r_), and then it remains constant. At this stage, the rear edge depins from its original position (sliding phenomenon) and droplet length remains constant. It can be observed that, for different evaporation times, spreading and sliding occur at different rotation speeds. More specifically, for bacteria-laden droplets with no evaporation (Figure 9-b, dark blue curves), only spreading occurs at lower rotation speeds, while a combination of spreading/sliding happens at later times. Comparable results are obtained for the case of the negative control. In this case, the advancing contact angle is about 53° and it is reached at 40 rpm. Droplet sliding is achieved after 50 rpm (which is in agreement with the results reported^31^ for comparable droplet volume i.e., 30 μL), with a receding contact angle of 15°.

Similar behaviors are observable under lower evaporation times (i.e., 15 and 30 min, Figure 8a,b, red and green curves), where a slight effect of cterial droplet barely sevaporation time is observed and is mainly associated with a delay in the inception of spreading and sliding stages (appearing at rotation about 10 rpm higher), although the final advancing and receding contact angle values are almost unvaried. This is true for both examined liquids. After 45 min, the effect of the evaporation is more pronounced. Microbe-laden droplets show an average initial contact angle of 35° (Figure 9b, dark pink curve), where the average initial contact angle is 25° for the case of uncontaminated droplets (Figure 9a, pink curve). Interestingly, as the rotation speed increases, it seems that the bacterial droplet barely spreads along the force direction but it never depins its rear edge from its initial location. Under this condition, droplets start spreading at about 55 rpm, suggesting that at higher evaporation times bacterial cells are mostly accumulated at the edges, whereas only a small amount of dark bulky liquid advances along the force direction. As the rotation speed further increases, the rear edge of the droplet stays pinned and the rear contact angle decreases until it becomes 15° at about 65 rpm. From that speed on, the dark bulky liquid part gradually squeezes toward the front edges leading to a smaller droplet length. After 70 rpm, the droplet abruptly disappears from the field of view. Similar behavior is observed at the highest investigated evaporation time (60 min, Figure 9b, dark violet curve), although, droplet depinning occurs at much higher rotation speeds and droplets start to squeeze later (about 80 rpm), where the droplet rear angle becomes 10° approximately and disappears from the scene after 90 rpm. Similar trends are obtained for 45 and 60 min-evaporated nutrient drops (Figure 9a), although different evaporation rates induce slight variations in terms of critical rotation speeds where droplet spreading begins. Droplet length vs rotation speed (Figure 9c,d) also shows differences as a function of evaporation times. The general trend is that, at the given evaporation time, droplet length increases due to the application of centrifugal forces. As the evaporation time increases, droplets are heavily stretched in the force direction, suggesting that higher retention forces are needed to obtain the onset of droplet sliding. To the best of our knowledge, this is the first time that evaporation/forced wetting of microbial droplet are explored.

The results can be compared with those of Hennes et al.,^28^ where *B. subtilis* droplet depinning on tilted agar substrates was investigated. In the latter case, active depinning is achieved due to the “*collective surfing*” motion induced by various mechanisms (i.e., production of biosurfactants and bacterial motility) overcoming capillary forces. In the present work, evaporation plays an important role in modulating droplet pinning/depinning behavior.

It is possible that, during the evaporation period, bacterial cells start attaching to the surface and secreting exopolysaccharides or adhesive proteins, promoting surface adhesion, in particular, in the case of higher evaporation times. An alternative explanation for the differences could be related to the different cell concentrations obtained after different evaporation times. To verify this hypothesis, droplets with different cell concentrations (from OD_600nm_ = 0.5 to 20 cm^–1^) not subject to preliminary evaporation are investigated. Front and rear contact angles and droplet length vs rotation speed are reported in Figure S2 (Supporting Information). Forced wetting parameters are not significantly affected by microbial concentrations and the general trend observed is comparable with that of water on pure glass. This confirms the hypothesis that the partial cell adhesion reached during the evaporation lag time, plays a key role in the phenomenon. Moreover, bacterial force adhesion is also influenced by the cell state as suggested by Raj M et al.^31^ Indeed, for alive cells, adhesion force reduces as a function of cell concentration, while for dead ones it increases.^30^ In this study, the retention forces needed for sliding increase at high evaporation times. Nevertheless, the specific effect of the cell physiological conditions cannot be hence identified. Therefore, further investigation concerning bacterial cell motility will be the subject of future works.

### Sliding Retention Force Determination

**Figure 10:**
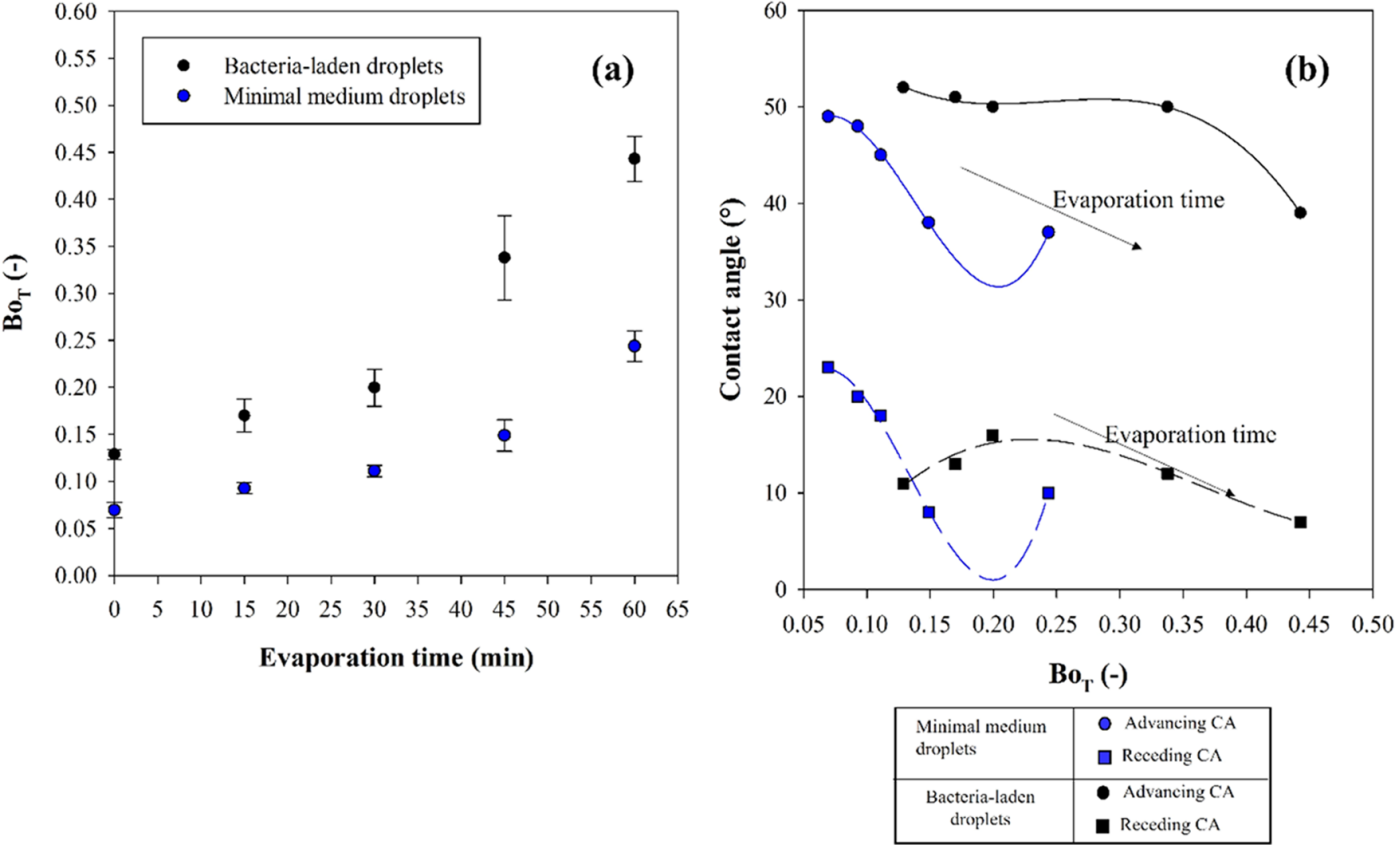
(a) Tangential bond number as a function of the evaporation times for bacteria-laden droplets (black curves) and minimal medium droplets (blue curves). (b) Advancing and receding contact angles as a function of the tangential bond number critical for sliding onset for minimal medium (blue curves) and bacteria-laden droplets (black curves).

The force needed for the onset of sliding is estimated by calculating the dimensionless tangential bond number (eq 2). For this calculation, it is assumed that, the density of the minimal medium is that of pure water at 25 °C, while, for the case of the bacterial suspensions, density is experimentally calculated (i.e., by determining the ratio of liquid mass over volume) and it is equal to 1200 ± 70 kg/m^3^. The surface tension values are assumed equal to 72 mN/m^53^ and 69 mN/m^54^ at 25 °C for minimal medium and *P. fluorescens*-laden suspension, respectively, assuming that they do not change during the evaporation process. For each condition, the critical tangential bond number is estimated at the rotation speed, at which droplets slide or disappear from the field of view. In Figure 10a, the critical Bond number as a function of the evaporation time is reported for the two examined liquids. Data are reported as the average and standard deviations of three independent measurements. In Figure 10b, the advancing and the receding contact angles of droplets exposed to different evaporation times are reported as a function of the tangential bond number needed for droplet sliding onset. Experimental data are shown as scatter points, whereas the cubic function of the experimental data is displayed by solid lines.

It is observed that the tangential bond number for droplet sliding onset is higher for denser bacterial droplets as evaporation time increases and always higher with respect to the minimal medium droplets, at a given time (Figure 9a). In addition, significant differences in the advancing and receding contact angles trend as a function of the critical tangential bond number for droplet runoff can be observed at different evaporation times (Figure 10b). Specifically, in the case of the negative control, the two angles soon decrease as the bond number increases, while the evaporation continues. For bacteria-laden droplets, advancing and receding contact angles strongly reduce only at the end of the experiment i.e., at 60 min, where the maximum value of Bond number is obtained.^35^

The critical tangential acceleration can be directly derived from the critical tangential bond number. A simple multiplication of the critical tangential acceleration by the droplet mass leads to critical tangential (equal to adhesion) force, *F*_s_. On the other hand, the tangential force depends on the selected characteristic length of the droplets, which is associated with the droplet shape. In order to isolate the effect of the microorganism on such force, it is decided to estimate the retention *k*-factor reported in Furmidge′s equation considering the characteristic lengths, the initial length (*k*_1_), and the length when the droplet slides or disappears from the field of view (*k*_2_), respectively, as already reported in previous publications^32^ and according to eq 3.
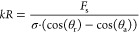
3where the retention force *F*_s_ is the applied tangential force at the moment of sliding inception, the values for the advancing θ_a_ and receding θ_r_ contact angles are taken as the average of experimental measurements. *R* is a length scale.

It is also known from ref (33) that droplet length represents the best choice for the calculation of the *k*-retention factor for either axial-symmetric or non-axial-symmetric droplets. Results are displayed in Table 1 for both minimal medium and bacteria-laden droplets, respectively.

**Table 1:** Values of the Retention Force Factor, *k*, Obtained from Two Different Characteristic Lengths, as a Function of the Evaporation Time for Minimal Medium and Bacteria-Laden Droplets

	minimal medium droplets				bacteria-laden droplets			
evaporation time (min)	initial length (mm)	*k*_1_ (-)	sliding length (mm)	*k*_2_ (-)	initial length (mm)	*k*_1_ (-)	sliding length (mm)	*k*_2_ (-)
0	6.1	1.8	6.3	1.8	6.0	2.5	7.3	2.0
15	6.1	1.2	6.5	1.2	6.0	3.2	7.1	2.7
30	5.8	1.4	6.0	1.4	5.9	2.3	7.1	1.9
45	5.4	1.1	5.9	1.0	5.8	2.8	6.3	2.6
60	5.2	1.1	5.7	1.0	5.4	5.8	6.4	4.9

*k*_1_ and *k*_2_ present comparable values either selecting the initial droplet length or the one at sliding, for both the examined fluids. More precisely, for the case of minimal medium, *k* is approximately kept constant to 1 along the evaporation time, achieving a comparable value with the case of water on pure glass under centrifugal forces.^33^ For the case of bacteria-laden droplet, *k* values are in the range of 2–6, where, the densification of bacterial load, induced by evaporation, leads to higher *k* values as evaporation time increases and therefore higher retention forces for the inception of depinning.

## Conclusions

In the present work, the effect of evaporation on the wetting behavior of bacteria-laden droplets on glass substrates is systematically investigated for the first time. Both evaporation and forced wetting are characterized in detail using a custom-made device named *Kerberos*. Comparison with uncontaminated control liquids is reported. Investigation of the internal droplet passive/active flows (i.e., chemotaxis and intrinsic bacterial motility) as well as subsequent pattern deposition is not taken into account in this work. Bacteria-laden droplets are found to be more resilient to evaporation with respect to droplets containing only nutrient medium. In addition, it is verified that at low evaporation times bacteria-laden droplets show similar properties with respect to water–glass systems where spreading and sliding can be distinguished. On the other hand, at higher evaporation times, higher cell accumulation at droplet edges occurs, to leave from the field of view, in analogy with what was observed in the case of pure water droplets on biofilm-coated glass surfaces. Dimensionless Bond number is calculated to quantify the retention forces needed for the onset of sliding, which is an indirect way of estimating bacterial adhesion forces. More quantitative results are also attained using Furmidge′s equation. The *K*-retention force factor is evaluated for selecting either the initial length or sliding length as characteristic scales. For both the examined fluids, comparable *k*-factors are observed. It is found that, for a given wetting agent, as the evaporation time is increased, higher retention forces for sliding inception are needed. However, at a given evaporation time, it is observable that the retention forces needed for sliding onset of bacterial droplets are higher than those of minimal medium droplets. In addition, no effect of the cell concentration on forced wetting properties is found. During evaporation and before forced wetting experiments, the live/dead assay is performed to check the physiological state of bacteria-laden droplets, whereas, both the two population cell densities increase over time, attaining steady-state conditions after 45 min.

This study can provide useful information for understanding bacterial adhesion and biofilm formation on surfaces which are certainly of interest for the design of antimicrobial coatings impairing cell attachment and in optimization of cleaning solution formulation. Possible future development of this work will include the use of other solid substrates investigating the role of hydrophilicity and hydrophobicity, as well as the effect of specific surface coatings. A dedicated study of bacterial motion within evaporating droplets will also be needed. Extension to bacterial strains with different phenotype/genotype features would also be of interest.

## Supporting Information Available

Repeatability checks in terms of front and rear contact angles and droplet length of bacteria-laden droplets at two evaporation times (Figure S1) and front and rear contact angles/droplet length vs rotation speed for different bacterial concentrations without taking into account evaporation (Figure S2) (PDF)

## Abbreviations

DLVO: Derjaguin–Landau–Verwey–Overbeek
CA: contact angle
RS: rotation speed
rpm: rounds per minutes
BoT: tangential bond number
