# Supplementary material for: Forced Wetting Properties of Bacteria-Laden Droplets Experiencing Initial Evaporation
Source: Langmuir. 2023 Apr 20;39(25):8589–602. doi: 10.1021/acs.langmuir.3c00179 (PMC10308807; doi:10.1021/acs.langmuir.3c00179)
Supplement: Supplementary file 1 — la3c00179_si_001.pdf [file la3c00179_si_001.pdf]

Forced wetting properties of bacteria-laden droplets experiencing initial evaporation

*Federica Recupido [1], Maria Petala [2], Sergio Caserta [3,4, *] Daniele Marra[3], Margaritis Kostoglou [1] and Thodoris D. Karapantsios [1#]*

1. Division of Chemical Technology, School of Chemistry, Aristotle University of Thessaloniki, University Box 116, 54 124 Thessaloniki (Greece).
2. Department of Civil Engineering, Aristotle University of Thessaloniki, University Box 10, 54 124 Thessaloniki (Greece).
3. Department of Chemical, Materials and Industrial Production Engineering (DICMaPI), Piazzale V. Tecchio 80, 80125, Naples (Italy).
4. CEINGE Advanced Biotechnology, Gaetano Salvatore 486, 80145 Naples (Italy).

Supplementary information

S1. Repeatability check

Repeatability check in term of front and rear contact angles and droplet length for bacteria-laden droplets is provided in Figure S1 for 30 min (A) and 45 min (B), respectively.

In both cases, consistency of measurements is attained, resulting in a maximum variation of ± 5 °. The droplet length shows different initial values among the repetitions, although the trend is repeatable.

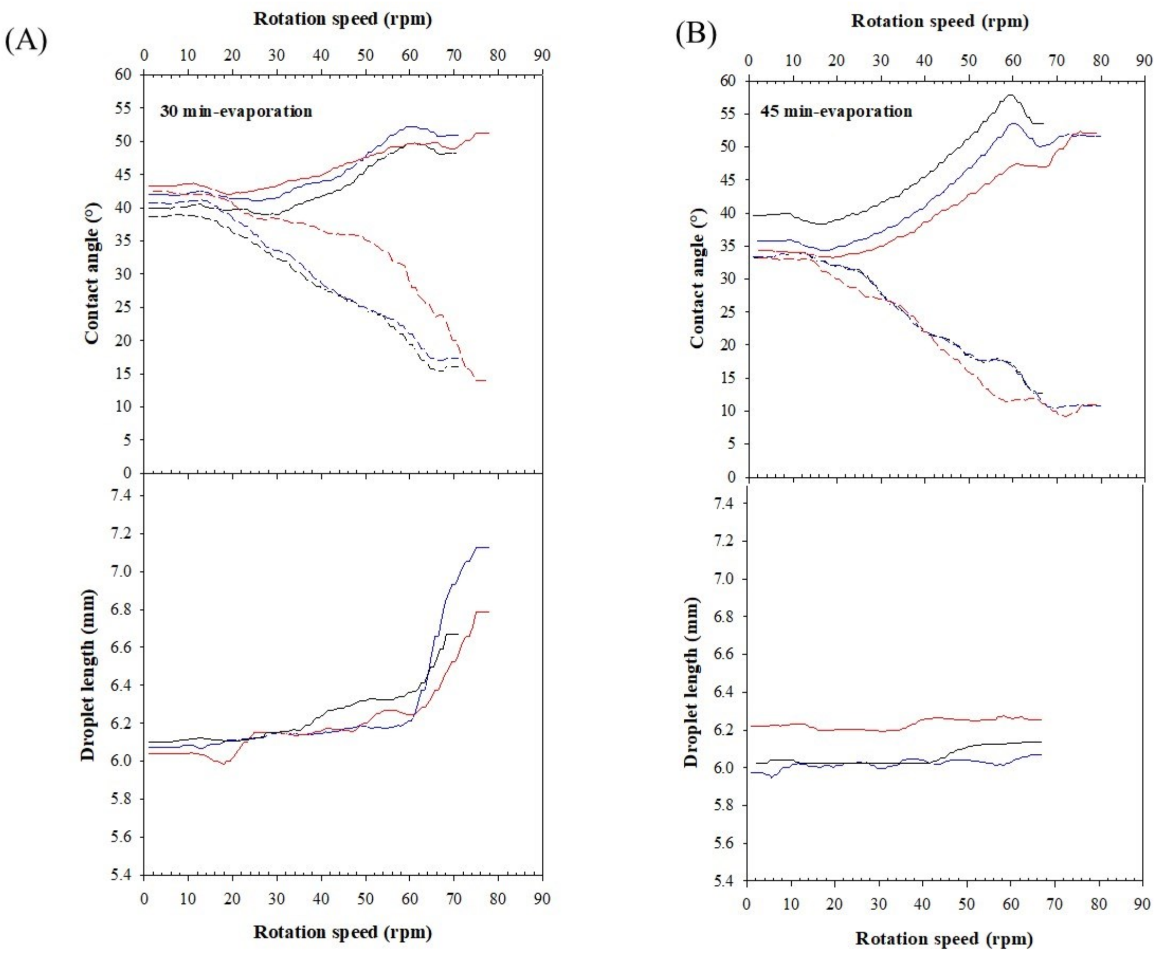

Figure S1. Front (straight lines) and rear (dashed lines) contact angle and droplet length vs rotation speed of three repetition of the same experiments (target rotation of 80 rpm, speed increase rate of 1 rpm/s, horizontal substrates) for bacteria-laden droplets undergoing to 30 min (A) and 45 min (B) evaporation.

S2. Forced wetting properties at different bacterial concentrations with no evaporation

In Figure S2 forced wetting properties of bacteria-laden droplets at different initial concentrations without undergoing evaporation process, are shown in terms of front and rear contact angles (a) and droplet length (b) as a function of the rotation speed.

Results demonstrate that no significant effect of the initial concentration is noticeable as in all cases, spreading as well as sliding are obtained at comparable rotation speeds.

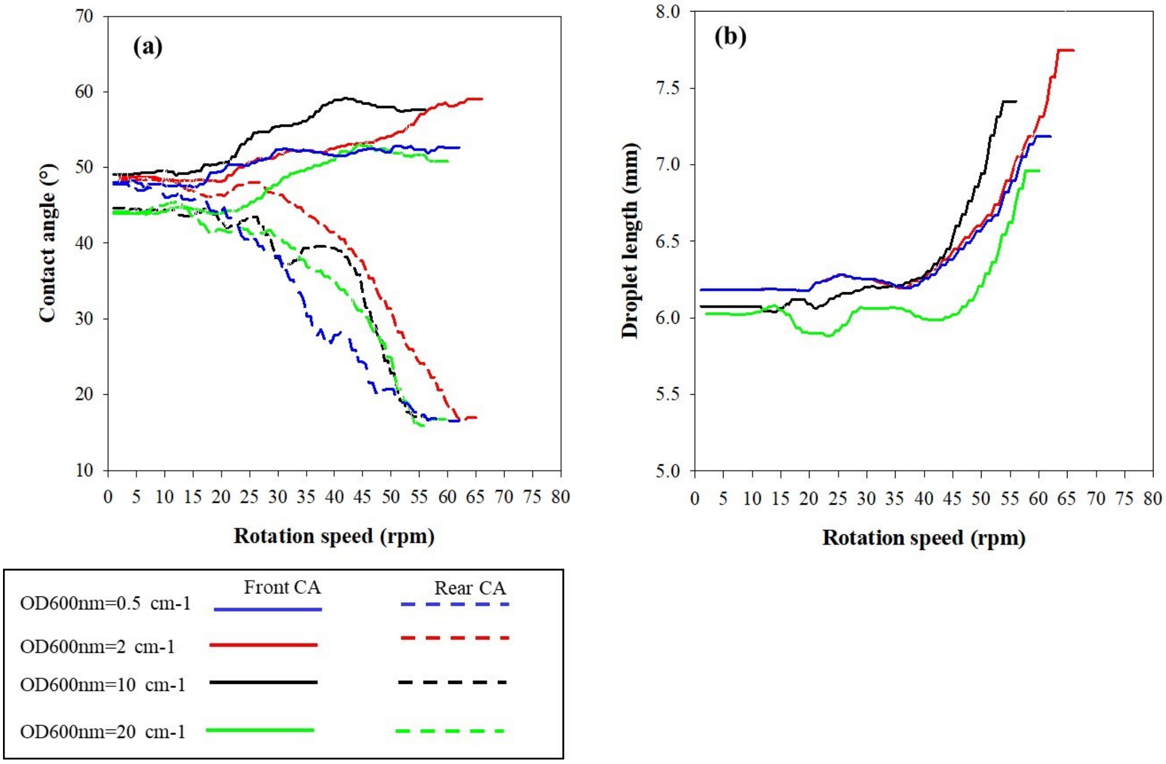

Figure S2. (a) Front (solid lines) and rear (dashed lines) contact angle and b) droplet length vs rotation speed of bacteria-laden droplets at different concentrations without evaporation.
